# Quality indicators for cervical screening in Sweden

**DOI:** 10.1177/09691413251362597

**Published:** 2025-07-31

**Authors:** Helena Andersson, Sara Nordqvist Kleppe, Henrik Edvardsson, K. Miriam Elfström, Joakim Dillner

**Affiliations:** 1 Center for Cervical Cancer Elimination, Pathology and Cancer Diagnostics, Medical Diagnostics Karolinska, 59562 Karolinska University Hospital, Stockholm, Sweden; 2 Center for Cervical Cancer Elimination, Department of Clinical Science, Intervention and Technology, 27106 Karolinska Institutet, Stockholm, Sweden; 3 Pathology and Cancer Diagnostics, Medical Diagnostics Karolinska, 59562 Karolinska University Hospital, Stockholm, Sweden; 4 Cancer Screening Unit, 679750 Regional Cancer Center of Stockholm-Gotland, Stockholm, Sweden

**Keywords:** Quality indicators, cervical cancer, screening, human papillomavirus, HPV, cancer prevention

## Abstract

**Objectives:**

Comparisons and optimization of screening programs are based on quality indicators (QI), but these are not well standardized and commonly not published. We report 13 QIs used for cervical screening in Sweden. These are decided on by the authorities and reported by the Swedish National Cervical Screening Registry that collects all data and calculates the QIs. The QIs as well as trends discovered and observations made during recent years, including effects of the COVID-19 pandemic, are summarized.

**Setting:**

Sweden. All units involved in cervical screening export all individual data to the screening registry.

**Methods:**

All data on screening invitations, cervical samples with human papillomavirus (HPV), cytology and histopathology results, as well as population data, cervical cancer cases and mortality, were collected. The 13 QIs were calculated.

**Results:**

The HPV screening test had a high population coverage of 83%, with most primary screenings using self-sampling. Follow-up for women with CIN2+ (cervical intraepithelial neoplasia) in cytology within 3 months was 60%, increasing to 95% or more after 1 year. The incidence and mortality of cervical cancer have decreased in recent years. Some QIs became outdated due to program changes, and there was significant variability between regions.

**Conclusions:**

The population coverage of the HPV screening test was not affected by the cancellation of screening appointments during the pandemic, because of switching to primary self-sampling. Improving follow-up of screen-positives and boosting population test coverage using HPV self-sampling are key areas for potential improvement.

## Introduction

Organized cervical screening can reduce mortality from cervical cancer by up to 90%.^1^ Most European countries have introduced a cervical screening program, albeit some of them are not population-based.^2^ Comparisons of screening programs, including identifying areas that need improvements, is possible if the screening data are collected in accountable population-based screening registries. The Swedish National Cervical Screening Registry (NKCx, www.nkcx.se) collects all data on cervical screening in Sweden with the main aim of measuring 13 quality indicators (QIs) for cervical screening decided on by the Swedish authorities (the National Board of Health and Welfare).^3^ The QIs had been in use by the NKCx for several years before they were formalized by the authorities in 2018. They were based on the European guidelines for quality assurance in cervical screening plus a dialogue with the regional screening program organizers in the country.^4^ The data are evaluated annually to inform the program of whether changes are needed in order to improve the benefits and reduce the harms of screening. The annual registry report details the 13 QIs. The QIs of the screening program for the years 2012–2016 have previously been published.^5,6^ Here, we summarize the key findings during 2017–2024 related to the QI measurements, including the impact the COVID-19 pandemic had on the screening program.

## Patients/methods

All laboratories in Sweden that perform human papillomavirus (HPV) analyses and cytological and histopathological diagnostics on samples from the cervix provide all individual data to the NKCx. Data files are exported from the laboratory information systems to the registry annually. Information on all cervical samples is collected. HPV samples include those taken by caregivers as well as self-samples, and tissue samples are not limited to the cervix but include all gynaecological localizations. Population data records are retrieved from the Swedish National Tax Authority, and invitations to screening are collected. Data on cervical cancer cases and mortality are collected from the National Board of Health and Welfare. The coverage for all data is 100%. The collected data are processed as previously described.^5^ Currently, the registry contains data on 24,800,781 invitations to cervical screening, 25,526,118 cervical samples analysed with cytology, 4,198,027 cervical samples analysed with HPV and 1,905,925 cervical tissue samples analysed with histopathology. Altogether, 5,216,974 women have at least one entry in the database. The Strengthening the Reporting of Observational Studies in Epidemiology (STROBE) cohort reporting guidelines were used.^7^

## Results

### Quality indicator 0: target population for invitation to the screening program

The National Board of Health and Welfare recommends that all women between the ages of 23 and 70 are invited to cervical cancer screening. The target population for invitation to screening is defined as all women between 23 and 70 years old who are resident in Sweden minus the women who have registered their opt-out of the screening program as well as women who do not have a cervix (total hysterectomies and women born without a cervix). The target population has been around three million women during the studied time period.

### Quality indicator 1: proportion of eligible women actually invited

QI 1 is the number of women who have been invited during the calendar year divided by the number of women in the target population (as defined in quality indicator 0) minus the number of women in the target population who already have taken a cervical HPV test within the recommended screening interval. This procedure is called ‘sorting out’ and is intended to avoid double testing of women who have already been screened at their own initiative (opportunistic screening).

We present this indicator for two different age groups (with different screening intervals) and for the year 2023. An invitation is considered to have been sent on time if it is sent less than 3 months after the recommended screening interval. QI 1 was 97.7% for age group 23–50 and 95.3% for age group 51–70. During 2020, there was a decline in the proportion of eligible women invited, due to cancellation of screening appointments caused by the COVID-19 pandemic. For example, in the large Stockholm region, the number of invitations in 2020 decreased to 64,641 compared to 115,292 invitations during 2019.

### Quality indicator 2: population test coverage within a screening interval

**Figure 1.**
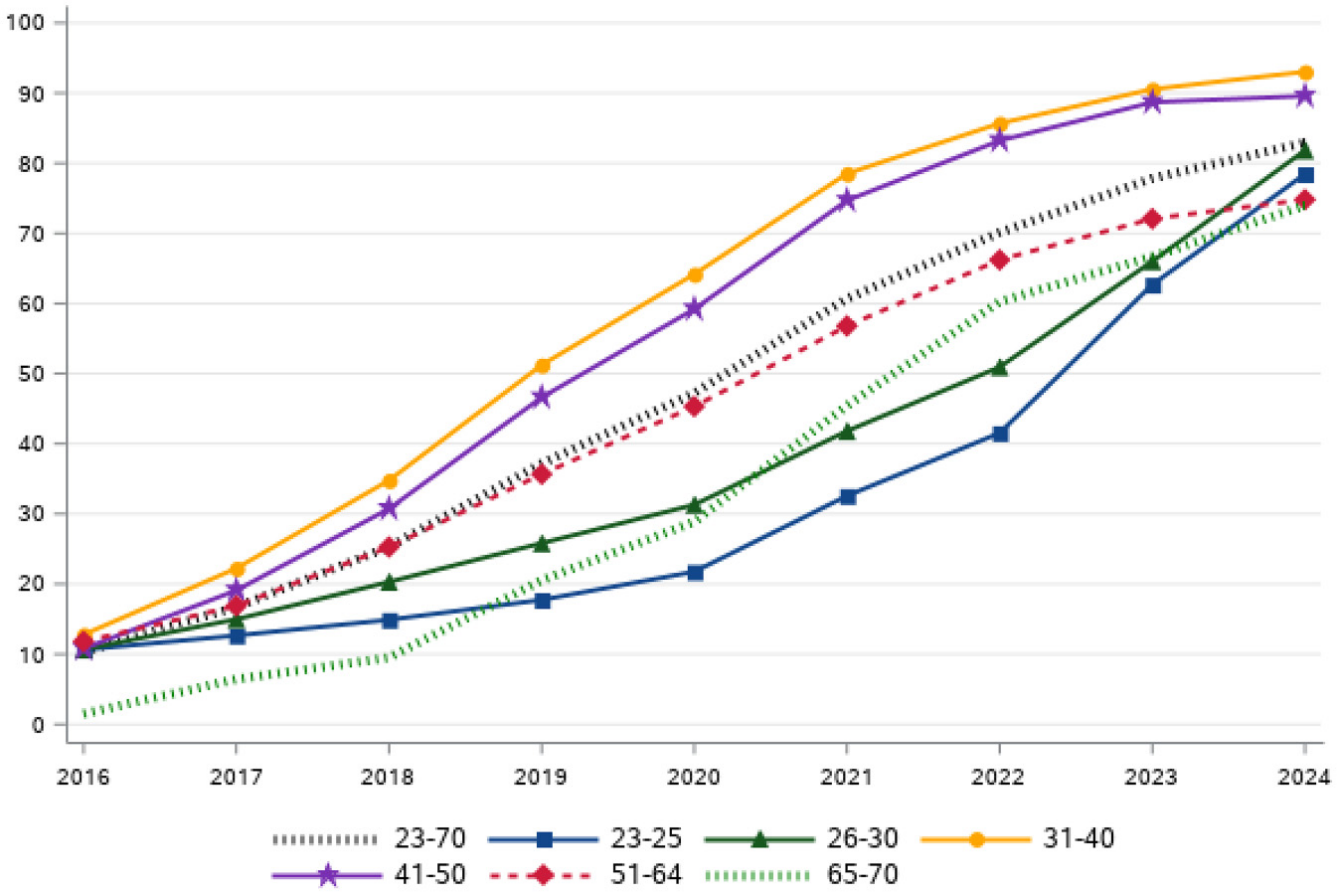
National test coverage (%) by age group during 2016–2024. The test coverage is increasing steadily in all age groups as illustrated by the black dotted line.

QI 2 is the number of women who have had at least one cervical sample taken within their recommended screening intervals, divided by the total number of women in the population in the target age group. Observe that the denominator here is the total population (also including the women opting out or without a cervix; see also QI 0). The numerator includes all cervical samples independent of whether the sample was taken within the organized screening program, by opportunistic screening or for some other reason. In the 2015 screening program, women aged 23–30 were offered cytology at 3-year intervals, women aged 30–50 were offered HPV testing at 3-year intervals and women aged 50–70 were offered HPV testing at 5-year intervals. In addition, there was a double test with both HPV and cytology at age 42.^5,6,8^ In the 2022 screening program, women aged 23–50 are offered HPV testing at 5-year intervals and women aged 50–70 at 7-year intervals. Since 2022, cytology is no longer counted as a screening test.^8^ The 2024 HPV test coverage by region and age group is displayed in Table 1. The development of the HPV test coverage over time, according to the 2022 screening program, is displayed in Figure 1.

**Table 1.** Population HPV test coverage (%) by region and age group in 2024.

	Age group			
Region	23–50	51–64	65–70	23–70
Stockholm	88.37	73.73	57.75	81.82
Uppsala	83.45	76.7	67.01	80.01
Södermanland	82.52	71.82	90.06	80.17
Östergötland	88.34	78.52	47.05	80.93
Jönköping	93.79	73.11	58.02	83.69
Kronoberg	74.94	67.04	71	72.2
Kalmar	92.96	83.55	86.98	89.15
Gotland	90.19	76.42	61.35	81.48
Blekinge	90.96	78.14	69.06	84.15
Skåne	88.31	79.11	80.63	84.93
Halland	97.12	80.34	97.89	92.01
Västra Götaland	85.32	69.98	85.12	80.95
Värmland	95.65	90.32	81.4	92.13
Örebro	94.59	70.56	82.76	86.28
Västmanland	91.64	80.87	99	89.93
Dalarna	94.42	83.81	47.68	84.78
Gävleborg	81.75	74.99	92.96	81.08
Västernorrland	90.13	75.91	68.25	82.76
Jämtland	92.15	65.42	60.58	80
Västerbotten	92.73	60.79	69.34	81.04
Norrbotten	85.21	65.62	86.55	79.13
**Sweden**	**88.37**	**74.8**	**73.96**	**82.93**

Bold values indicate that the row displaying the numbers for the as a summary of the upper rows.

About 75% of all cervical samples were taken by the organized, invitational screening program. The number of non-organized samples varied strongly between regions, from one region with almost all samples being organized (98%) to regions where only about half of all samples were organized (Table 2). The quality assurance programs are, however, the same for non-organized and organized testing.

**Table 2.** Number of cervical samples and proportion of organized cervical samples in Sweden 2017–2023.

Year	2017		2018		2019		2020		2021		2022		2023	
Region	Number of cervical samples	Proportion of organized cervical samples (%)	Number of cervical samples	Proportion of organized cervical samples (%)	Number of cervical samples	Proportion of organized cervical samples (%)	Number of cervical samples	Proportion of organized cervical samples (%)	Number of cervical samples	Proportion of organized cervical samples (%)	Number of cervical samples	Proportion of organized cervical samples (%)	Number of cervical samples	Proportion of organized cervical samples (%)
Stockholm	158,799	69	160,823	69	178,983	74	112,243	56	223,700	74	213,606	76	166,667	79
Uppsala	27,198	56	29,885	57	28,806	62	27,735	69	34,426	74	29,216	68	28,361	81
Södermanland	17,031	76	19,900	79	18,397	77	19,718	81	25,351	87	16,986	98	14,249	98
Östergötland	33,134	73	33,518	76	35,471	80	30,461	76	34,742	78	34,581	79	36,733	81
Jönköping	25,715	77	29,936	80	26,610	80	25,640	80	33,254	66	32,986	75	24,300	78
Kronoberg	12,962	68	15,229	71	17,645	69	15,006	43	24,668	46	20,028	44	22,747	44
Kalmar	17,749	76	20,469	75	19,506	76	17,209	78	19,022	78	19,268	79	18,313	75
Gotland	3871	68	3533	67	3962	77	2710	56	4761	70	4388	75	3800	72
Blekinge	10,052	68	11,358	77	12,178	63	11,614	53	11,787	70	9085	66	14,683	81
Skåne	124,753	65	99,159	67	111,849	74	99,868	72	112,340	74	113,855	73	126,543	72
Halland	24,515	72	22,073	77	28,511	83	27,925	85	25,988	81	26,822	84	19,043	79
Västra Götaland	139,074	69	142,288	70	139,321	77	125,933	76	153,652	80	131,565	78	106,419	60
Värmland	27,294	66	22,746	66	22,235	70	23,732	75	24,736	79	21,676	74	27,515	77
Örebro	20,377	75	25,160	84	25,638	84	19,140	77	21,385	78	19,696	75	19,659	77
Västmanland	20,375	76	16,153	73	23,162	85	17,906	79	22,886	83	23,203	74	21,607	85
Dalarna	22,563	76	26,178	80	25,361	77	20,903	73	23,458	78	24,784	80	14,480	70
Gävleborg	22,357	75	22,536	77	21,506	77	18,913	70	19,922	75	21,634	83	29,856	85
Västernorrland	18,222	80	15,656	78	19,405	82	18,731	82	21,953	84	18,313	82	18,181	79
Jämtland	9380	73	8530	72	10,285	71	9951	74	11,285	83	11,160	93	9698	80
Västerbotten	19,055	71	19,529	72	23,132	76	21,170	76	23,780	83	21,347	78	22,304	83
Norrbotten	19,880	78	18,602	78	16,746	78	12,601	77	19,959	87	17,426	85	15,733	85
**Sweden**	** 774,356**	**70**	**763,263**	**72**	**808,709**	**76**	**678,609**	**71**	**893,055**	**76**	**831,625**	**76**	**760,891**	**75**

Bold values indicate that the row displaying the numbers for the as a summary of the upper rows.

### Quality indicator 3: number of women participating in screening following an invitation

QI 3 is a quality measure of the efficiency and accessibility of the organized cervical screening. The attendance rate remained high throughout the period with an attendance rate after invitation of around 70%. This QI is nowadays combining the proportion of invited women who show up for an appointment with the number of women who are sent a self-sampling kit to their home address and do return a sample.

Self-sampling has been recognized as an effective approach to cervical screening and a preferred option in particular for women who have not attended screening.^9–12^ Invitations to screening by healthcare personnel and sending of self-sampling kits are considered equal alternatives in Sweden.

During the COVID-19 pandemic, all non-emergency healthcare including screening was almost completely stopped. The Swedish National Board of Health and Welfare allowed for screening with primary self-sampling for HPV instead of clinician-based sampling in all age groups.^13^ As a consequence, the use of self-sampling increased dramatically from 2020 to 2021, and in 2023, 58% of all self-sampling was done within the screening program (Table 3). The pandemic thus did not affect the attendance rate.

**Table 3.** Number and percentage of self-samples taken within the organized screening program of all self-samples taken, per year and region.

Year	2020			2021			2022			2023		
Region	In total	Screening		In total	Screening		In total	Screening		In total	Screening	
	Number	Number	Proportion (%)	Number	Number	Proportion (%)	Number	Number	Proportion (%)	Number	Number	Proportion (%)
Stockholm	5666	149	2.6	130,893	90,535	70	140,976	85,476	61	53,023	34,374	65
Östergötland	996	118	11.8	9338	7358	79	17,320	13,857	80	17,867	12,832	72
Jönköping	259	81	31.3	0	0	0	0	0	0	36	1	3
Skåne	12,089	5892	48.7	39,783	29,346	74	60,652	39,893	66	66,928	43,237	65
Västra Götaland	648	255	39.4	18,509	14,050	76	31,944	27,488	86	33,120	26,204	79
Örebro	0	0	0	0	0	0	484	59	12	854	82	10
Gävleborg	0	0	0	0	0	0	382	15	4	798	71	9
Jämtland/Härjedalen	0	0	0	62	9	15	996	80	8	692	471	68
Västerbotten	0	0	0	1743	155	9	290	67	23	1571	1176	75
Kronoberg	0	0	0	0	0	0	0	0	0	142	30	21
Kalmar	0	0	0	0	0	0	0	0	0	221	103	47
Halland	0	0	0	0	0	0	0	0	0	15	7	47
Elimination project^a^	NA	NA	NA	NA	NA	NA	9130	2385	26	55,831	14,742	26
All regions	19,658	6495	33.0	200,328	141,453	71	262,194	169,327	65	231,098	133,330	58

a National campaign with concomitant HPV vaccination and HPV screening in the age group born between 1994 and 1999. All women in the project are HPV tested also if they have already been HPV screened. Thus, only 26% of these samples are at the timepoint when the women were due for screening.

### Quality indicator 4: proportion of women with abnormal screening results

QI 4 enumerates the number of women whose cervical sample tested HPV positive, divided by the total number of samples that have been HPV tested with adequate results. The indicator reflects the prevalence of HPV in the population. The proportion of participating subjects with a non-normal result is an essential QI for screening programs, to estimate both the resources required for follow-up and the magnitude of harms. The percentage of HPV-positive women in HPV primary screening has been around 10% in the age group 30–70. HPV prevalence was formerly high among women younger than 30 years but is no longer common among young women because of the high coverage of the school-based HPV vaccination program.^14^ In 2012, school-based HPV vaccination began for girls born in 1999 or younger and became gender-neutral in 2020. HPV vaccination coverage was 82–83% among women born in 1999–2000. There was a 98% decline in HPV-16 prevalence and a 99% decline in HPV-18 prevalence among the 2000-born women compared to the 1984-born women.^14^ The strong effect of vaccination on the HPV prevalences necessitates that this QI needs to be re-measured every year.

### Quality indicator 5: proportion of inadequate samples for HPV analysis

This indicator describes the number of cervical samples tested for HPV but with an inadequate result, divided by the total number of samples tested for HPV. This QI is a measure of the quality of the sampling. During the last 6 years, this QI has consistently been <0.5%.

### Quality indicator 6: proportion of cervical samples without endocervical cells

The lack of endocervical cells in a cervical sample has traditionally been used as an indicator of the sampling quality. However, our registry linkages found no association at all between lack of endocervical cells and cancer risk,^15^ questioning whether lack of endocervical cells should be reported at all because of the risk of focusing the quality work on irrelevant indicators. Also, as cervical cytology is no longer classified as screening, neither as a primary test nor as a triaging method for HPV16-/18-positive women, measurement of this QI is less relevant to the screening program and will most likely be discontinued. At any rate, the finding has been rather common, found in about 6–8% of all cervical samples tested with cytology.

### Quality indicator 7: percentage of women with normal cytology after a positive HPV test

QI 7 measures the precision of the currently recommended triaging method (cytology is the recommended triaging method among HPV-positive women). The percentage of samples with normal cytology after an HPV-positive test is around 50% throughout the period. However, in our randomized healthcare policy trial, it was shown that HPV16-/18-positive women with a normal cytology triage test were at extremely high risk for cervical cancer, questioning whether triage testing with cytology is safe.^16^ Another argument against the use of cytology in triaging is that some laboratories report very high proportions of cytological atypia in almost all cases of HPV triaging. In these laboratories, triaging with cytology does not work as almost all women are referred to colposcopy. Therefore, results from cytology are no longer used for triaging of HPV16-/18-positive women, and QI 7 is likely to be replaced by a new QI evaluating a new triaging process in the future.

### Quality indicator 8: percentage of women with an abnormal screening result that are followed up

The effectiveness of a screening program largely depends on the follow-up of abnormal screening results. A measurable proportion of women with invasive cervical cancer have had cell changes that have not been followed up.

QI 8 has so far been based on follow-up of abnormal cytologies, i.e. high-grade intraepithelial lesion (HSIL) or worse. The number of women with HSIL+ per year varied between 8614 and 11,230 during the period (Table 4). The proportion of HSIL+-positive women who were followed up with histology within the recommended follow-up time (3 months after the cytology result) has been low, 59–68%. However, 1 year after the diagnosis, 95–97% of the HSIL+-positive women had been followed up. Similar to the attendance rate, the follow-up time was not affected by the COVID-19 pandemic (Table 4). There is large regional variation with the proportion of women varying between 20–86% and 68–99% within 3 and 12 months, respectively. In the future, this QI will be changed to measure the proportion of HPV-positive women who have been followed up.

**Table 4.** Follow-up with histology after CIN2+ (HSIL) in cytology.

		% women with follow-up histology			% women with follow-up histology			% women with follow-up histology			% women with follow-up histology			% women with follow-up histology			% women with follow-up histology			% women with follow-up histology	
Region	No. of women with CIN2+ (HSIL+) 2016	Within 3 months	Within 1 y	No. of women with CIN2+ (HSIL+) 2017	Within 3 months	Within 1 y	No. of women with CIN2+ (HSIL+) 2018	Within 3 months	Within 1 y	No. of women with CIN2+ (HSIL+) 2019	Within 3 months	Within 1 y	No. of women with CIN2+ (HSIL+) 2020	Within 3 months	Within 1 y	No. of women with CIN2+ (HSIL+) 2021	Within 3 months	Within 1 y	No. of women with CIN2+ (HSIL+) 2022	Within 3 months	Within 1 y
Stockholm	1953	85	97	2320	84	98	2587	80	98	2563	79	97	1922	81	97	1873	78	97	2147	75	98
Uppsala	289	73	97	294	71	98	370	41	96	401	64	98	382	71	97	361	59	98	259	53	98
Södermanland	150	80	95	216	65	97	268	68	98	255	71	96	209	72	95	268	51	75	158	32	68
Östergötland	481	84	93	421	82	97	460	66	97	550	66	98	451	84	98	350	73	99	251	69	97
Jönköping	263	70	96	279	64	98	357	55	98	411	58	98	401	67	97	421	61	95	283	56	95
Kronoberg	166	59	93	115	48	96	141	54	99	209	51	99	166	74	98	182	77	98	120	80	96
Kalmar	314	86	99	369	77	98	385	80	98	321	71	96	300	76	97	245	80	97	190	69	98
Gotland	57	80	96	65	84	94	65	79	97	77	82	96	71	76	99	46	74	98	57	69	98
Blekinge	163	66	98	79	63	96	132	50	97	172	69	99	136	73	99	161	49	98	132	62	98
Halland	209	68	98	243	73	96	199	71	97	260	76	97	385	80	96	314	71	94	251	60	94
Skåne	1445	58	92	1507	52	90	1548	58	91	1461	60	91	1210	62	94	1242	62	98	1373	58	96
Västra Götaland	1835	49	96	1730	36	75	1682	49	95	1637	54	95	1509	68	97	1422	67	95	1417	65	97
Värmland	468	65	98	437	69	98	361	71	98	307	73	99	384	78	98	299	80	98	257	80	96
Örebro	266	55	97	286	69	98	303	65	99	351	65	97	296	59	98	299	57	98	285	66	98
Västmanland	182	78	99	207	67	99	186	64	98	210	58	92	189	69	96	188	49	97	155	39	97
Dalarna	199	76	95	344	79	99	581	65	97	947	55	96	489	64	96	394	62	96	326	69	92
Gävleborg	142	64	90	169	63	97	159	73	98	134	67	94	158	76	97	179	64	97	179	63	97
Västernorrland	229	52	98	266	66	97	268	63	97	320	43	97	257	54	98	279	62	96	205	62	94
Jämtland	174	21	99	116	20	96	118	26	97	143	36	95	115	27	99	152	20	94	140	29	98
Västerbotten	458	45	97	402	57	95	351	62	97	284	67	96	246	64	93	266	56	96	236	49	93
Norrbotten	203	78	99	327	62	98	242	61	98	217	48	99	157	52	97	211	47	95	193	40	95
**Total**	**9646**	**66**	**97**	**10,192**	**64**	**96**	**10,763**	**62**	**97**	**11,230**	**63**	**96**	**9433**	**68**	**97**	**9152**	**62**	**96**	**8614**	**59**	**95**

CIN: cervical intraepithelial neoplasia; HSIL: high-grade intraepithelial lesion.

### Quality indicator 9: proportion of women with normal cytology after a positive HPV test that have a follow-up sample taken within recommended time period

Women with an HPV-positive test result but where the following cytology is normal should be followed up with HPV and cytology double testing within 18 months for HPV16/18, 36 months for medium oncogenicity HPVs and 60 months for low oncogenicity HPVs. The proportion of HPV16/18+/cytology-negative women who are actually followed up has improved over time. For 2022, 70.7% had taken a follow-up sample within the recommended interval of 18 months, and 99.9% had taken a follow-up sample within 24 months.

### Quality indicator 10: incidence of cervical cancer in a screening interval after a negative screening result (interval cancer)

This indicator enumerates the women who have received a cervical cancer diagnosis within a screening interval (plus half-a-year) after a normal screening test result. This is the only QI that directly measures the protective effect of the screening against the cancer itself. The women who have taken a test and received a normal result have a reduced risk of cervical cancer. Of course, no tests are completely fail-safe, and cancer can occur before the next screening. It is important to quantify the incidence of interval cancer as a key QI of the screening test itself.

The false-negative cytologies, i.e. samples with normal cytology but where cervical cancer was diagnosed within a screening interval (interval cancer), rose over time from 17 to 25 false-negative samples per 100,000 samples between 2008 and 2015.^17^

### Quality indicator 11: waiting time from sample registration to notification of test results

This indicator is measured in days and should be as short as possible to reduce anxiety after sampling. There is also evidence that an extended waiting time and thereby delayed management increase the risk that a precursor has progressed to cervical cancer.^18^ Since the sampling date cannot be determined with certainty in the case of self-sampling, the registration date at the laboratory is used instead for all samples. The median number of days in 2023 for the whole country was 6, but the variability among laboratories was large, ranging from 0 to 29 days.

### Quality indicator 12: incidence of cervical cancer

To measure the ultimate outcome of the screening program, the prevention of cervical cancer, the number of newly discovered cervical cancers per 100,000 women aged 0–84 is monitored. The incidence rates have been age-standardized with the Swedish population in 2000 as the standard population. Data on invasive cervical cancer have been retrieved from the National Board of Health and Welfare’s statistical database and data on the average female population from the statistics database of Statistics Sweden. This QI can spot an up-going or down-going trend in cervical cancer incidence. The age-standardized incidence of cervical cancer was found to be highest during the period 2017–2019 in the whole country (11.29) as well as in most regions. In some regions, including the Stockholm region, the incidence continued to increase during the period 2020–2022 (from 10.52 to 11.07/100,000 in Stockholm). However, the incidence in the entire country decreased during the same period (10.53). The full dataset on cervical cancer incidence is available in the NKCx annual report for 2024, which can be found at www.nkcx.se.

### Quality indicator 13: cervical cancer mortality

QI 13 describes the mortality per 100,000 women in the age range 0–100+. This QI is calculated using the data from the National Cause of Death Register. It was found that cervical cancer mortality varies greatly from year to year and between different regions. This is because of the relatively few cases and few inhabitants in some regions, where one single case can strongly affect the outcome. As an example, for the year 2020, the mortality rate in Sweden was 1.93, to be compared to 13.3 worldwide or 2.2 in Northern Europe per 100,000 women.^19^ In several regions, a lower mortality rate during 2021–2022 was observed, which correlates with the introduction of HPV testing as a primary analysis method.

## Discussion

### Statement of main findings

We report on the national QIs of an organized cervical cancer screening program over time, measured using a comprehensive, individually identifiable national screening registry. The most obvious conclusion is that changes in the screening program are making several QIs outdated, making international comparisons difficult. For instance, as cytology is no longer considered a screening test in Sweden and women tested with cytology are classified as unscreened, screening coverage based only on HPV tests cannot be compared to the screening coverages in countries that still consider women tested with cytology as screened.

Strong findings include high HPV test coverage and the fact that, despite the cancellation of screening appointments in healthcare during the pandemic, the population test coverage remained high, due to the switch to primary self-sampling for HPV. Areas for improvement include the low follow-up rate for screen-positive women (about 60% of women with HSIL+/CIN2+ in cytology were followed up within the mandated 3 months; see Table 4). The low rate is not a registry artefact, as >95% of these women were found to be followed up after more than 1 year. In another setting, using computer simulation, it was found that if all eligible women get full screening and follow-up tests recommended for them, 23% of the cervical cancer cases could be prevented, emphasizing that there is a lot to be gained by improving follow-up routines.^20^ Regarding population test coverage, the rate of follow-up of screen-positive women and whether invitations had indeed been issued to eligible women, there were striking regional disparities that need to be addressed to ensure consistent care. Regional disparities also draw attention to inequities in screening. A clear definition of how equity should be measured as a QI is lacking, but to evaluate equity in screening would probably require several QIs to measure the impact of screening for underserved populations in different steps of the screening process. To be able to evaluate the screening also requires that variables defining certain underserved groups are registered, which is not always the case. Another aspect of screening that is not covered by the current QIs is the potential harm of, for instance, overscreening in terms of physiological impact, physical harms and financial costs, of which at least some are readily measurable. This has been pointed out by the Swedish National Board of Health and Welfare in the updated national screening guidelines.

### Strengths of the study

The NKCx has 100% national coverage of data on invitations, cell samples and tissue samples from the cervix. The data are accessible for research after approval from the Swedish Ethical Review Authority. The completeness and accessibility of the data from the cervical screening program in combination with well-defined QIs decided on by a government agency make the evaluation of the screening program robust, transparent and comparable over time. The study is of conceptual importance and displays what can be done under the right conditions, having access to complete datasets and structured evaluation criteria.

### Weaknesses of the study

A striking finding is that the changes to the screening program, in particular the screening test used, the triage test used and the use of self-sampling, made several of the decided QIs obsolete. Comparisons with other countries and with older screening programs are therefore not straightforward.

### Comparison with other studies

In a systematic review from 2023, five different guidelines for cervical cancer screening could be identified that recommended nine different performance quality metrics for cervical cancer screening that are related to QI 5, QI 10 and QI 12 in this report. None of the guidelines, however, provided evidence to support the recommended test performance metrics.^21^

The lack of evidence to support the use of specific QIs is a shortcoming of the whole field, including for the QIs described here. However, this study could contribute to describing their feasibility and perceived usefulness. A similar study to the one presented here was performed in Denmark in 2017 using data on the screening population from Statistics Denmark.^22^ In addition, data on cervical screening was retrieved from the annual reports from 2009 to 2015 on screening quality published by the Danish Quality Database for Cervical Screening (DKLS). Results corresponding to QI 2 (test coverage) and QI 8 (percentage of abnormal and unsatisfactory samples not followed up within recommended time intervals) were included. It was concluded that although the key QIs can provide a basis for improvement of a screening program, they do not explain the regional differences in incidence of cervical cancer that were seen in Denmark at that time. When comparing QIs from all quality registries for screening in Denmark, it was found that of all 27 analysed, the Danish Quality Database for Cervical Cancer Screening was the registry that fulfilled the lowest proportion of indicator standards of all registries.^23^ Only one of the seven indicators was fulfilled once during a 3-year period on a national level. This could either mean that the quality of the screening program is deficient or that the QI thresholds are set on an unfeasibly high level. The fact that the same level of fulfilment of indicators was seen when looking at the regional level, meaning that not a single region could meet the standards, argues for the latter.

This also highlights the difficulty in setting the thresholds for QIs. What would be a reasonable threshold for a QI and how to decide that threshold? On the one hand, if the threshold is set too low, apart from the risk of accepting insufficient quality, an increase in quality over time would pass unnoticed, since the quality would always be fulfilled according to the QIs. On the other hand, if the threshold is set too high, the quality over time might improve but would not be reflected in fulfilled QIs, since the threshold would never be reached.

In a report from NHS England, the performance of the cervical screening program in England during 2021 and 2022 was compared to legacy data and in relation to 12 standards.^24^ Data were reported both at national and regional levels. Although several standards were not met at a national level, in part due to the aftermath of the COVID-19 pandemic, they were in most cases met in at least some regions of the country. This implies that the QI thresholds were set at a feasible level. Depending on how a cervical cancer screening program is organized and reported, the opportunity to be able to compare national units, where at least some units are able to meet the quality threshold, could give legitimacy to the indicator threshold. Comparison between countries where the organization of the screening program and the available infrastructure are similar could also be a source for setting the desired aims of the QIs.

The WHO has launched a global strategy for the elimination of cervical cancer.^25^ The strategy includes targets to be reached by 2030 dealing with cervical cancer incidence, vaccine coverage, screening participation and treatment of precancerous lesions. These targets could provide thresholds for at least four indicators.

Not only do thresholds for QIs vary between countries, if at all available, but also the kind of indicators used to measure the performance of a screening program. In the Swedish setting, focus is on coverage of invitations and tests, laboratory analyses, follow-up and final outcomes, whereas others also include vaccine coverage and treatment,^26^ assessment^27^ or screening outcome.^28^

The European Union CanScreen-ECIS project has worked out a set of 23 performance indicators based on all potential existing indicators found in the literature for quality assessment of cancer screening programs. The indicators were rated based on importance and feasibility. The ones deemed most important were found in categories covering detection rates, examination coverage and interval cancer rate.^29^ The CanScreen-ECIS performance indicators could be a starting point for a more harmonized evaluation system for cancer screening.

## Conclusions

Compiled data from the NKCx for the period 2017–2024 is presented in relation to 13 key QIs. The main aim of a cervical screening program is to reduce the incidence of and mortality from cervical cancer. Thus, QI 12 and QI 13 can be regarded as the final outcome QIs of the program. Major areas for improvement include better follow-up of screen-positive women and reduced regional disparities in QIs. Future, additional QIs evaluating inequities and harms of screening should be considered.

The incidence of cervical cancer in Sweden started to increase during 2014, which was found to be caused by the declining protection from normal cytology tests.^30–32^ However, the incidence is now declining, both in the country as a whole and in several regions, coinciding with the switch to screening using the HPV test instead. There are clear signs of a downward trend at the end of the reported period (www.nkcx.se). The QIs recommended in Sweden do not come with any threshold values (except QI 2, which is supposed to be >85%). Therefore, the quality improvement of the screening program is monitored by comparing the QIs over time.

### Implications

In August 2020, the World Health Assembly adopted the Global Strategy for cervical cancer elimination, which is defined as maintaining an incidence rate of cervical cancer below 4 per 100,000 women.^25^ The current incidence rate in Sweden is 11.15/100,000. The mission to eliminate cervical cancer in Sweden is realistic within a few years from now, but this will require that the tools provided are further sharpened and used as intended. The purpose of cervical cancer screening programs is to intercept the development of cervical cancer. The purpose of QIs is to serve as tools for evaluating the quality and performance of the screening program and drive quality improvements of the program. An annual evaluation of the screening programs by compiling QI measurements and comparing them with legacy data can guarantee that cervical cancer elimination is proceeding in the right direction. The results of the QI reports are shared with the medical guideline development groups, providing insights into the implementation of recommendations in everyday clinical practice. This feedback stimulates the ongoing development of the corresponding guidelines. As cervical cancer screening is continuously evolving with, for instance, new screening methods and follow-up strategies, the QIs may need to change to monitor and assess the performance of the screening program. By presenting the outcomes of a national screening program in the international literature, the results can inform other cancer screening programs also challenged with how to achieve as fast an elimination of cervical cancer as possible.
